# Neurobiological Trajectories Involving Social Isolation in PTSD: A Systematic Review

**DOI:** 10.3390/brainsci10030173

**Published:** 2020-03-18

**Authors:** Ilias I Vlachos, Charalambos Papageorgiou, Maria Margariti

**Affiliations:** 1st Department of Psychiatry, Eginition Hospital, National and Kapodistrian University of Athens, 115 28 Athens, Greece; chpapag@med.uoa.gr (C.P.); mmarg@med.uoa.gr (M.M.)

**Keywords:** PTSD, social isolation, neuroendocrine/neuroimmune reaction, neuroplasticity, pharmacological targets, telomere length

## Abstract

Social isolation (SI) stress has been recognized as a major risk factor of morbidity in humans and animals, exerting damaging effects at the physical and mental health levels. Posttraumatic stress disorder (PTSD), on the other hand, occurs as a result of experiencing serious, life-threatening, traumatic events and involves involuntary re-experiencing trauma (intrusion), avoidance symptoms, and distortions of cognition and emotional arousal. The literature shows that PTSD is affected by genetic predisposition and triggers a large neurocircuitry involving the amygdala, insula, hippocampus, anterior cingulate- and prefrontal-cortex, and affects the function of the neuroendocrine and immune systems. Social isolation seems to influence the predisposition, onset and outcome of PTSD in humans, whereas it constitutes a valid model of the disorder in animals. According to the PRISMA (preferred reporting items for systematic reviews and meta-analyses) protocol, we systematically reviewed all original studies involving the neurobiological trajectories between SI and PTSD published till July 2019 (database: PubMed/Medline). Out of 274 studies, 10 met the inclusion criteria. We present the results of the retrieved studies in terms of hypothalamic-pituitary-adrenal (HPA)-axis and endocannabinoid system function, immune reactions, neuroplasticity, novel pharmacological targets, and shortening of telomere length, which confirm a synergistic effect on a neurobiological level between the two entities.

## 1. Introduction 

Posttraumatic stress disorder (PTSD) is a chronic psychiatric condition resulting from direct or indirect exposure to serious trauma (death, threatened death, actual or threatened injury, or sexual violence) and involving intrusive reexperiencing of the traumatic event in the form of unwanted memories, nightmares, flashbacks, emotional and physical distress after exposure to traumatic reminders, persistent avoidance symptoms to trauma-related stimuli, negative alterations of cognitions and mood (dissociative amnesia, negative beliefs about oneself, negative emotional state like fear, horror, guilt and inability of experiencing positive emotions), as well as marked alterations of arousal and reactivity related to the traumatic event (irritability, anger, aggression, self-destructive behavior) [1]. The lifetime prevalence of PTSD in the general population according to the Diagnostic Statistical Manual (DSM)-5 criteria is around 8% with women scoring higher than men [2], whereas the prevalence of the disorder among high-risk groups, like war-veterans, may be four times higher [3]. The risk for PTSD increases with the number of traumatic experiences [4], whereas more than 80% of PTSD patients present one or more comorbidities like depression, substance use, and physical health issues which lead to debilitating outcomes [5,6] as well as increased mortality rates [7], rendering, hence, the understanding and treatment of the disease a complex crossroads for the clinician. Multiple studies have shown that trauma-related disorders are associated with the dysfunctioning of numerous biological systems, and that PTSD symptom severity exerts a cumulative effect on premature aging of the immune system and telomere length [8].

Human functional imaging studies have identified an increased connectivity between the ventromedial prefrontal cortex (vmPFC), locus coeruleus (LC), hypothalamus, hippocampus, basolateral amygdala (BLA), and bed nucleus of the stria terminalis (BNST) in PTSD patients, while more subcortical areas like the periaqueductal gray area (PAG) and central amygdala (CeA) gradually become involved when the severity of a threatening stimulus becomes aggravated [9,10].

Before the last decade, the predominant neurobiological model of PTSD concentrated on the pathway of fear conditioning involving mainly the amygdala system [11]. The preparations of the new DSM-5 broadened the criteria to include further emotionally dysregulated states like anger, guilt, shame, and symptoms of derealization and depersonalization, as well as altered self- and other-related conditions and deficits in social cognitions. Such changes also gave rise to new neurobiological research challenges [12]. The emotional dysregulation in PTSD tends to be viewed as a vulnerability factor relating to genetic and developmental parameters and a response to (a) traumatic event(s) during childhood, adolescence or adulthood [11]. Neuroendocrine and neuroimmune findings in PTSD include lower basal cortisol output, increased glucocorticoid receptor (GR) function, and a proinflammatory response pre-, peri-, and post-trauma [13], whereas early trauma [14] and ongoing threat [15] are associated to similar observations. The endocannabinoid system (e-CB) has also been related to the pathophysiology of stress-related psychiatric disorders as PTSD. It includes the main e-CBs: anandamide (AEA) and 2-arachidonoylglycerol (2-AG) that act on receptors type 1 (CB1) and (CB2). The CB1 receptor is widely expressed in the prefrontal-limbic system [16] and, along with anandamide, it positively modulates fear extinction acting mainly in the hippocampus and basolateral amygdala [17]. N-palmitoylethanolamine (PEA), an endogenous lipid modulator, is considered part of the extended e-CB next to AEA and 2-AG is considered an analgesic, neuroprotective and antioxidant agent, exerting its pharmacological function by stimulating the peroxisome proliferator-activated receptor -α (PPAR-α), a ligand-activated nuclear receptor [18]. Research has shown that PEA levels in blood show strong negative association with PTSD symptom severity in humans [19] and become increased by antidepressants in corticolimbic regions of rats [20]. Furthermore, PEA binding at PPAR-α interacts with the gamma-aminobutyric acidergic (GABAergic) neurosteroid system in the biosynthesis of allopregnanolone (Allo) [18]. Allo is a positive allosteric modulator of GABA action at GABA_A_ receptors and regulates emotional behavior by exerting anxiolytic, antidepressant, sedative effects. It is produced from progesterone in glutamatergic neurons of the cortex, hippocampus and basolateral amygdala through the double enzymatic action of 5α reductase type I (5 α-RI) and 3 α-hydroxysteroid dehydrogenase(3α-HSD) [18,21]. The decrease of Allo in the cerebrospinal fluid of PTSD patients correlates to the severity of reexperiencing and depressive symptoms of both men and women. Interestingly enough, the block in the biosynthesis of Allo is sex-specific relating to 5 α-RI deficiency in men and to 3 α-HSD deficiency in premenopausal women [22,23]. In animal models of PTSD, including the isolated mouse, corticolimbic downregulation of Allo levels correlates to enhanced contextual fear and impaired fear extinction, implying that this neurosteroid may serve as a useful biomarker and treatment option for PTSD across humans and animals [21].

Different models have tried to explain the psychoneurobiological etiology of stress-related mental disorders and their shaping interaction with trauma(s): The three-hit concept of vulnerability and resilience [24] developed by Daskalakis et al. focuses on the interplay between genetic predisposition, early-life adverse events and later-life environment on the occurrence of psychopathology: According to this testable hypothesis, early mild to moderate stress in animals may contribute to stress inoculation and hypothalamic-pituitary-adrenal (HPA) axis adaptability to later adverse events, whereas more severe early stress may affect, beyond HPA-axis functioning, amygdala activation and processing, fear retention, as well as vulnerability to later-life stressors and stress-related disorders (like PTSD) in adulthood. This model does not follow the linearity of the “classic” cumulative stress model (or stress diathesis model), where additive accumulation of different stressors, that exceed a certain threshold, leads to the development of psychopathology to vulnerable individuals, but offers instead an evolutionary counterpart of the dynamic interchange between genetics (hit-1) and early-life events (hit-2) affecting endocrine function and epigenetic modifications, shaping in turn an evolving phenotype which may adapt and resist (resilience) or succumb to mental disorders (vulnerability) when faced with later life stressors (hit-3).

Social bonds play an important role in mediating psychological and physical well-being [25]. Social isolation (SI) stress constitutes a major risk factor of morbidity and mortality for humans as well as nonhuman social species [26]. Research in animals has shown that SI exerts a burdening effect on animal physiology [27], increasing oxidative stress, proinflammatory cytokines, basal cortisol levels, as well as the occurrence of obesity and type II diabetes, urinary catecholamine levels, thus influencing immunity, inflammation control, and genes regulating glucocorticoid responses in a negative way. If post-weaning SI rearing follows maternal separation (reminding us of the three-hit concept of vulnerability), the metabolic risk is multiplied, and rats exhibit up to a 120% increase of fasting glucose compared to group-housed animals [28]. Other study has shown that prior SI in prairie voles alters concentrations of monoamine neurotransmitters following acute restraint, with isolated animals exhibiting elevated serotonin and dopamine levels in the hypothalamus and potentially decreased levels of serotonin in the frontal cortex [29]. Long-term isolated rats also showed a stronger activation of the sympatho-adrenomedullary system (SAS) when faced with new stressors compared to rats chronically exposed to long-term crowded conditions [30]. Neonatal isolation may also increase social dominance and aggression in rats compared to non-isolated controls, resembling the aggressiveness of people who have experienced early neglect. Research has found that neonatal isolation stress increased the stable fraction of actin, which is glucocorticoid dependent and these altered actin dynamics at the spines in the juvenile mPFC may explain neocortical dysfunction leading to altered social behavior later in life [31]. Moreover, in another study, the SI of adolescent mice rendered them unable to forget aversive memories when tested one month after the original event and fear memory retention was explained by the increase of brain-derived neurotrophic factor (BDNF) in the hippocampus [32].

Last but not least, SI is considered a valid animal model of PTSD in the sense that socially isolated animals present symptoms that resemble the human form (face validity) of the disorder like anxiety behavior, contextual fear responses, impaired fear extinction, cognitive alterations, aggressiveness, and neuroendocrine changes [33]. These disturbances are attributed to the corticolimbic downregulation of allopregnanolone (Allo), which normally exerts fast allosteric modulation of the action of GABA at the GABA_A_ receptors, as mentioned before [21].

In humans, the stress of SI refers to the subjectively perceived feeling of isolation or “loneliness” which does not always coincide with objective social isolation or social support. Neurobiological studies are fewer for obvious reasons, involving more peripheral biological variables: Loneliness has been correlated to a variety of medical conditions, like elevated blood pressure, increased HPA activity, metabolic syndrome, as well as Alzheimer’s disease progression [34]. The upregulation of proinflammatory cytokines during acute stress has, moreover, been observed among the lonelier participants of a study involving healthy adults and breast cancer survivors, implying immune system dysregulation [35]. Loneliness may also predict subsequent depressive symptomatology that cannot be attributed to other parameters [34]. Solitary confinement or disciplinary isolation is a penal tool used in the prison system of the U.S. and other countries against the most violent of the inmates. Segregated individuals display higher levels of mental distress compared to the rest of the general prisoner population and manifest a wide range of psychiatric symptoms ranging from anxiety, panic attacks, and depression, to psychotic symptoms, self-mutilation, or even suicide. Another part of isolated inmates become even more violent after this punitive measure. These detrimental effects of disciplinary segregation have led in the U.S. to calls for the reform of solitary confinement, especially for juvenile offenders, given the irreversible damage that can be caused on their still developing brains [36]. A lack of social support is considered a major risk factor for PTSD following traumatic events [37], while the presence of social support seems to influence symptom severity and recovery [38]. PTSD patients may not make use of social support in order to protect others from distress, thinking that others will not understand or for fear that they will undermine their own self-image [39]. Withdrawal and real or expected negative responses from others relating to early experiences of bullying victimization may cultivate a vicious circle of shame and loneliness in these patients [40]. In addition, the neurobiology of social support is still poorly understood [11].

Given the referred interchange between SI stress and PTSD and in the absence of a systematic review, we wanted to illuminate in this paper the neurobiological trajectories between the two entities and the degree to which the interaction with SI aggravates the (neuro)biological progression of PTSD.

## 2. Methods

### 2.1. Search Strategy

The literature search was conducted in the electronic databases PubMed/Medline. The database search was carried out between May and July 2019. There was no limit set regarding the publication year. The search strategy is exemplified for PubMed/Medline. The search terms were combined with the Boolean operator as follows: “social isolation” AND “PTSD”.

### 2.2. Eligibility Criteria 

Articles were included that met the following inclusion criteria: (a) animal and human original studies investigating the neurobiological and biological associations between SI and PTSD and with mentioning of both terms in their title, key words or text. In terms of publication status, articles in print or published ahead of print were accepted. The exclusion criteria were: (a) studies dealing with behavioral aspects of PTSD (description of symptomatology among different patient groups, development and use of diagnostic tools, psychotherapy, rehabilitation programs, (b) studies relating PTSD to organicity (head injury, cancer, myocardial infarction, chronic pain syndromes), (c) literature reviews, or (d) no available full text. 

### 2.3. Study Selection

Each study was screened for eligibility by the first author after reading the title and abstract. Any uncertainties concerning eligibility were discussed and resolved among all authors. The decisions for inclusion or exclusion are summarized in a flow chart according to PRISMA (preferred reporting items for systematic reviews and meta-analyses) recommendations. PRISMA is an evidence-based manual for reporting in systematic reviews and meta-analyses and focuses on the reporting of reviews evaluating randomized trials, but can also be used as a basis for reporting systematic reviews of other types of research [41].

## 3. Results

### 3.1. Process of Study Selection

**Figure 1:**
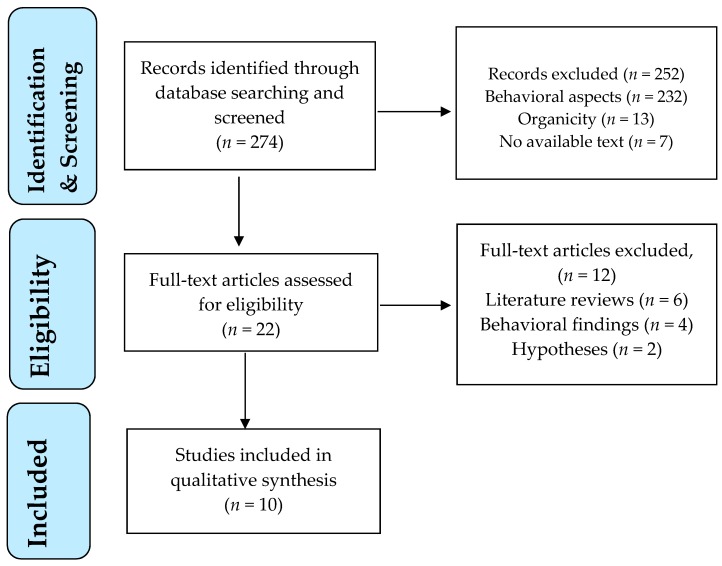
PRISMA flowchart of study selection process.

A flow diagram outlines the study selection process (Figure 1).

The electronic database search retrieved 274 publications (Figure 1). All titles and abstracts were screened for their relevance to the topic. Twenty-two articles were identified as potentially relevant after screening of titles and abstracts. These studies were assessed for eligibility in full text. Of these, 10 studies met the eligibility criteria and were included in the review, dating between 2008 and 2019. The main reasons for study exclusion were: inclusion of behavioral findings with no neurobiological variables and literature reviews or hypotheses without research confirmation.

### 3.2. Characteristics of Included Studies

Key characteristics of included studies are summarized in Table 1. 

All retrieved studies were published between 2008 and 2019. Eight studies concerned animal research and two investigated human subjects. Four out of the eight animal studies [42,43,44,45] used mice as experimental subjects and four studies [46,47,48,49] used rats, divided in socially isolated (experimental) and group-housed animals (controls). Face-validity [33,50] was established in all animal studies, since they phenomenologically resembled the human setting of PTSD with inescapable electric foot shock serving as the most common traumatic experience (male intruder, predator odor, restraint, and forced swimming were, moreover, used in parallel or independently). Construct validity [33,50] was served in terms of the effort to identify common underlying mechanisms with the human disorder, whereas predictive validity [33,50] was present in three of the animal studies [42,44,47] in terms of providing predictions concerning therapeutic responses and novel pharmacological targets. Hippocampal and amygdala involvement was investigated in seven of the studies [42,43,45,46,47,48,49], the role of the HPA-axis and its products in four studies [45,46,48,49], while the endocannabinoid system in three [44,47,48]. The immune profile of stressed animals and upregulation of proinflammatory cytokines was examined in one of the studies [45]. In the two human longitudinal studies conducted by the same research group [51,52], the telomere length of prisoners of war with PTSD was examined 42 years (T_2_) after war captivity (T_0_) with an intermediate survey of perceived social isolation (T_1_) 18 years after trauma occurrence.

In the following section, we will approach the neurobiological trajectories between social isolation and PTSD, presented as main findings in the reviewed studies in terms of HPA-axis and endocannabinoid system function, immune reactions, neuroplasticity, novel pharmacological targets, and shortening of telomere length. 

#### 3.2.1. Hypothalamic-Pituitary-Adrenal (HPA)-Axis Function

Total plasma [45,48,49] as well as adrenal CORT levels [46] were decreased in animals exposed to both SI and trauma compared to controls. In Cheng et al. [49] corticosterone levels were higher if compared to neonatally isolated (NI) animals with no stress exposure, but lower compared to the levels of animals exposed only to SPS and not neonatal isolation (NI). Increased pituitary ACTH release was observed as an immediate reaction to stress after social isolation, possibly associated with enhanced c-Fos expression in the amygdala [46,48]. However, a decrease in ACTH levels was observed six months after the incidence repeated unpredictable stress in previously isolated animals [46]. Upregulation of glucocorticoid receptors (GR) immunoreactivity was observed in the hypothalamus and the hippocampus [48,49] of SI animals and it was associated with stress-induced hippocampal neuronal loss and volume reduction. Mineralocorticoid receptors (MR), on the other hand, were downregulated and this observation was associated to a reduced resilience of isolated animals to new stressors [48].

#### 3.2.2. Endocannabinoid System (e-CB)

SI rats that were never exposed to extinction procedures after the electric shock experience were found to have decreased hippocampal anandamide levels (AEA) compared to rats that had followed extinction procedures after the traumatic experience in Morena et al. study [47]. Levels of endogenous AEA can be elevated by URB597, an inhibitor of AEA’s main degrading enzyme, FAAH. In a study by Boero et al. [48], SI increased hypothalamic AEA and CB1R concentrations, while reducing hypothalamic 2-AG levels that may be involved in HPA-axis negative feedback dysregulation.

#### 3.2.3. Proinflammatory Reactions 

Lower plasma corticosterone levels six months after RUS was associated with a two-fold increase of proinflammatory cytokines IL-1β and IFN-γ in socially isolated animals of the Algamal et al. study [45].

#### 3.2.4. Neuroplasticity

Brain plasticity also becomes affected by the combined influence of SI and traumatic exposure as relevant studies show. The dorsolateral bed nucleus of the stria terminalis (dlBNST), a subregion of the extended amygdala, plays a critical role in stress-induced plasticity by regulating HPA axis activity. It mediates between the corticolimbic system by receiving stressful stimuli and sending GABAergic projections to the paraventricular nucleus of the hypothalamus (PVN), where corticotrophin releasing hormone (CRH) is released and peripheral stress response gets started under pituitary activation [37]. Acute and chronic social isolation seem to provoke blunting of long-term potentiation in the dlBNST in the Conrad et al. study [43], compared to group-housed animals. Moreover, hippocampal BDNF and pro-BDNF in the amygdala of isolated animals were downregulated six months after RUS in the Algamal study [45]. Also, the expression of synaptic proteins was altered in conditions of combined NI and SPS with Synapsin1 being reduced in the basolateral amygdala and the hippocampal dentate gyrus, while PSDN95 was increased in both the hippocampus and amygdala, whereas the ratio between NLG1/NLG2 was significantly increased in the hippocampus and decreased in the amygdala of NI+SPS animals in the Cheng et al. [49] study.

#### 3.2.5. (Novel) Pharmacological Targets

SI mice present decreased corticolimbic levels of allopregnanolone (Allo) (an endogenous positive allosteric modulator of GABA action at GABA_A_ receptors) associated with the downregulation of 5α-reductase type I, the step enzyme in Allo biosynthesis [42,47]. The decrease of allopregnanolone is associated with altered emotional responses like aggression, increased contextual fear, and delayed contextual fear extinction. It has been shown that selective brain steroidogenic stimulants (SBSS) like fluoxetine and S-norfluoxetine may normalize Allo levels in the mPFC, hippocampus, and BLA and attenuate contextual fear responses related to social isolation [42]. Moreover, in the Locci et al. study [44], S-fluoxetine, along with the Allo analogs GNX, BR351, and BR297 that directly act at GABA_A_ receptors, as well as the endocannabinoid PEA that stimulates brain Allo biosynthesis were used to alleviate symptoms of aggression of isolated mice, as observed in the resident–intruder situation. PEA and BR297 proved to have a stronger anti-aggressive effect than S-fluoxetine [44]. Last but not least, URB597 can increase the levels of the endogenous AEA and is effective in enhancing fear extinction and promote social interaction [47].

#### 3.2.6. Shortening of Telomere Length

Telomeres are nucleoproteins located at the end of chromosomes protecting them from oxidative stress. Although not a neurobiological marker, telomere length (TL) is considered a substantial biomarker of cellular senescence, of immune system integrity and predictor of mortality [51], as well as a reliable peripheral biological measure of stress and hostility of PTSD patients [53,54]. In two retrieved studies, Stein et al. [51,52] showed that, in ex-prisoners of war (POW), solitary confinement during captivity as well as feelings of loneliness, loss of role in the family, lack of social support, and being accused at homecoming were associated with a shortening of telomere length in later life, compared to ex-POWs who did not share these experiences and feelings. A limitation of the study, as stated by the authors, is the small number of participants (*N* = 83, *N* = 99 respectively) and the long intervals between the examined periods.

Last but not least, the neurobiological findings of the above mentioned studies were mirrored in behavioral and cognitive symptoms of the stressed humans and animals including: fear responses [42,46], impaired fear extinction [42,45,47], increased anxiety behavior [33,45,49], aggression [44], as well as depression and feelings of loneliness for humans [51,52].

## 4. Discussion

Neurobiological research on PTSD and social isolation relies heavily on animals and deals mainly with peripheral biological variables in human subjects. Most of the retrieved studies showed a synergistic effect between PTSD and SI resembling to an aggravated version of neurobiological findings that comprise the broader PTSD literature as for example the key feature in many of the retrieved papers [45,48,49] concerning the blunting or hypoactivation of HPA-axis, already known from PTSD research [55]. Nevertheless, qualitatively different is the work of Daskalakis et al. [46] where a novel, isolating environment early in the life of maternally separated rats primes amygdala activation and an enduring fearful phenotype throughout life. In accordance with his three-hit concept of vulnerability [24], the author outlines a profile of fear perpetuation that is the result of social isolation and trauma, acquiring more permanent than transient characteristics.

The upregulation of proinflammatory cytokines in the Algamal study [45] is a more predictable and not pathognomonic finding, in the sense that general inflammatory responses across different psychiatric disorders can affect mood, behavior, and cognition [56]. In terms of neuroplasticity, the decrease of hippocampal BDNF levels and the retention of fear memory six months after the RUS paradigm in isolated rats [45] contradicts another study stating that social isolation of rats during adolescence led to hippocampal BDNF increase and retention of fear memories during adulthood [32] and further research is needed to clarify these opposing findings.

Concerning pharmacotherapy, the recent American Psychiatric Association (APA) and National Institute of Clinical Excellence (NICE) guidelines include antidepressants like the SSRIs fluoxetine, paroxetine, sertraline, and the SNRI venlafaxine, as well as the antipsychotics prazosin, olanzapine, and risperidone as first- and second-line agents for the treatment of PTSD [57]. Studies on the effectiveness of cannabinoids in the treatment of PTSD are small and disputed and better randomized clinical trials are needed [58] in order to corroborate the findings of the retrieved studies. The same holds true for the role of Allo in the treatment of PTSD combined with SI, that also needs further research and clinical support [59]. 

## 5. Limitations

The limitations of this review paper include the nature of most of the studies that focused on animal research, thus restricting translational limitations to the human condition. Even the two studies with the ex-POWs suffer from methodological limitations due to the small sample size, the long time-intervals between examination periods, and the retrograde distortion of memory concerning self-reports from the period of captivity [51,52].

## 6. Conclusions

SI exerts an aggravating influence on neurobiological consequences before, in parallel, or after trauma occurrence in PTSD, as the studies in this paper suggest. Despite the limitations of this review, it is important to retain that addressing social isolation, when trauma occurs, constitutes an essential secondary prevention measure. It would also be of interest for future research to further substantiate the pharmaceutical pathways of neurosteroids and endocannabinoids for the treatment of non-responders to usual agents by organizing well-designated clinical trials. Given the ubiquitous role of genetic predisposition, the three-hit concept of vulnerability [24] is justified in sketching the interplay between PTSD and social isolation. Further research is needed to illuminate this relation by exploring perhaps common biomarkers of allostatic load in human subjects across the two conditions [60,61]. PTSD patients constitute a group of vulnerable individuals whose trauma and the deriving biological and psychosocial repercussions have yet to be better understood and alleviated. 

## Figures and Tables

**Table 1:** Key Characteristics of Included Studies.

Study	Type	Neurobiological Measure(s)	SI Stress	PTSD Measure(s)	Main Findings
Pibiri F. et al. (2008) [42]	Isolated and group housed (groups of 5) *adult* male Swiss-Webster mice	-5aReductase Type I (5aRI) -Allopregnanolone (Allo) -S-norfluoxetine, selective brain steroidogenic stimulant (SBSS)	3–4 weeks of isolation of the experimental group	Inescapable electric foot shock (unconditioned stimulus) Acoustic tone (conditioned stimulus)	SI enhances contextual fear responses and impairs fear extinction and this finding relates to Allo and 5aRI downregulation in mPFC, hippocampus and amygdala. Allo downregulation may be reversed by S-norfluoxetine
Conrad K.L. et al. (2011) [43]	Isolated and group housed (*n* = 9–11) male C57Bl/6J mice (7–8 weeks old)	-dlBNST, subregion of extended amygdala serving as relay of corticolimbic information to paraventricular nucleus of the hypothalamus	-Acute SI (1 day) -Chronic SI housing (6–8 weeks)	Electrophysiological tetanus protocol	Acutely and chronically isolated animals presented blunting of dlBNST longterm potentiation compared to group housed animals. Moreover, they exhibited anxiety-like behavior in the novel open field
Daskalakis N.P. et al. (2014) [46]	Wistar rat pups (male/female till pnd9, after that only male rat pups) divided into four groups (*n* = 8–10 per group) (i) repeated maternal separation (MS) in home-environment (HOME-SEP), with the pups remaining together (ii) repeated-MS in a novel-environment (NOVEL-SEP), the pups were individually housed in a novel-environment; (iii) repeated handling of daily brief (15 min instead of 8 h) MS in the home-all together or in a novel-environment individually (HOME-HAN and NOVEL-HAN); (iv) no-separation/no-handling (NON-SEP/NON-HAN) control condition.	-Amygdala c-Fos expression -Pituitary ACTH-release -Adrenal CORT secretion	Prolonged isolation from siblings in a novel-environment during repeated-maternal separation between post-natal-day (pnd) 3–5	Contextual fear-conditioning on pnd 240 in a fear-conditioning box. Floor of the box consisted of stainless steel rods, connected to a shock generator. Rats were individually placed in the shock box. After 2 min, one electric foot shock was given and 2 min later, the rat returned to its homecage. Re-exposure: 24 h later, the same procedure was repeated however without delivery of the foot shock	The stress of prolonged isolation from siblings in a novel-environment during repeated-MS (NOVEL-SEP) activates the amygdala fear network with enduring consequences for behavioral and endocrine fear-reactions. NOVEL-SEP pups exhibited increased amygdala activation and pituitary ACTH-release that persisted from early-life into adulthood, while adrenal CORT-secretion was reduced.
Locci A. et al. (2017) [44]	Swiss-Webster male mice Isolated at pnd 25 Isolated at pnd 45	-S-fluoxetine-Selective Serotonin Reuptake Inhibitor (SSRI) -Allo analogs: ganaxolone, BR351, BR297 -PEA-endocannabinoid	Isolation at pnd21 (early adolescence) and at pnd45	Male intruder mouse was placed in a resident home cage and resident–intruder interactions were videotaped. aggressive behavior of SI mice was characterized by exploratory activity around the intruder, rearing and tail rattle, wrestling and/or a violent biting attacks.	Isolation of early adolescent mice induced more severe aggression than isolation later in life. all drugs had better effect on late onset aggression while S-fluoxetine had the lowest response rate in treating aggression, compared to endocannabinoid PEA with the highest response rate.
Stein J.Y. et al. (2018a) [51]	Longitudinal study 83 Israeli ex-POWs	Telomere Length (TL) measured at T_2_ (2015) utilizing the Southern Blot measured in total white blood cells obtained from 10 mL of blood	Loneliness and perceived Social Support measured at T_1_ (1991) with -UCLA-loneliness scale-Müller’s social network approach/depression measured with Symptom Checklist-90/PTSD measured with PTSD-Inventory (PTSD-I)	Captivity on the Egyptian and Syrian Fronts during the Yom-Kippur War in 1973 (T_0_)	Lack of perceived social support and loneliness at T_1_ were both negatively correlated with TL at T_2._ Depression was positively correlated with PTSD, lack of perceived social support, and loneliness. Lack of perceived social support and loneliness were positively correlated.
Stein J.Y. et al. (2018b) [52]	Longitudinal Study 99 Israeli ex-POWs	TL measured at T_2_ (2015) utilizing the Southern Blot measured in total white blood cells obtained from 10 mL of blood	At T_1_ (1991) self-reports of *captivity suffering* in terms of: (a) weight loss, (b) physical abuse, (c) solitary confinement *Homecoming experience* in terms of: (a) received social support (b) loss of place in the family (c) sense of accusation (d) loneliness	Captivity during the Yom-Kippur War in 1973 (T_0_)	When all the study variables were accounted for, solitary confinement in captivity and interpersonal factors at homecoming (i.e., loss of place in the family, sense of loneliness, sense of being accused by society at homecoming) significantly contributed to shorter TL
Morena M. et al. (2018) [47]	Sprague-Dawley male rats All rats individually housed Rats were divided to a group exposed to extinction procedure and rats never exposed to extinction procedure. One week after trauma, exposed to extinction rats were subjected to three spaced extinction sessions, mimicking human exposure therapy	-e-CB -AEA FAAH inhibitor URB597 increases AEA concentration	3 days individually housed before the experiment	5 inescapable footshocks	-Rats never exposed to the repeated extinction procedure presented decreased hippocampal AEA levels when measured immediately after the SI session URB597, through indirect activation of CB1 receptors, enhanced the consolidation of extinction, as well as ameliorated the trauma-induced alterations in social behavior by increasing the levels of social interaction.
Boero G. et al. (2018) [48]	Isolated and group housed (groups of 5) male Sprague-Dawley rats at pnd25	COR; CBG; CRH; ACTH; mMR; mGR; CB1R; AEA; 2-AG.	30 days of isolation (between pnd25–pnd55)	5 min of acute foot-shock stress at pnd55	SI decreased plasma total CORT and CBG concentration levels, however, after acute stress exposure, SI rats showed long-lasting CORT, ACTH and CRH responses indicating dysregulation of the HPA axis; SI also induced downregulation of hippocampal mMR and upregulation of hippocampal and hypothalamic mGR; Observed overexpression in GR mRNA was linked to stress-induced hippocampal neuronal loss; SI also affected the hypothalamic eCB system: compared to group-housed rats, basal levels of AEA and CBR1 were increased, while basal levels of 2-AG were decreased in socially isolated rats suggesting that social isolation alters eCB-mediated signaling in the hypothalamus, likely inducing an impairment in glutamatergic and GABAergic inputs that control CRH release, contributing to the impairment of the feedback inhibition of the HPA axis.
Algamal M. et al. (2018) [45]	C57BL/6 male mice (aged 8–10 weeks) stressed group (*n* = 17) and group housed (*n* = 15) (controls)	-Hippocampal volume; hippocampal BDNF and CRH; Plasma CORT levels- amygdala Pro-BDNF, its signaling receptor P75NTR and NMDA receptor 1; proinflammatory cytokines; hypothalamic FKBP51	Single housing for the experimental stress group of rats for the 21 days of the stress experiment and for the following 6 months till the end of the study.	Repeated unpredictable stress (RUS) paradigm involved 21 days of (i) daily unstable social housing with an alternate congener, (ii) unpredictable repetitive exposures to danger-related predator odor (fox urine, TMT), while restraint for 30 min, (iii) physical trauma in the form of five repeated inescapable footshocks, (iv) lack of social support, single housed post stress	6 months after RUS: stressed animals showed lower CORT and ACTH levels and reduced levels of FKBP51 in the hypothalamus, suggesting blunted HPA-axis reactivity; twofold increase of proinflammatory cytokines IL-1β and IFN-γ was observed; volume of the CA1 region of the dorsal hippocampus was significantly reduced and hippocampal BDNF levels were significantly downregulated; in the amygdala reduction in the levels of ProBDNF and P75NTR were observed. Stressed mice demonstrated recall of traumatic memories, passive stress coping behavior, acute anxiety, and weight gain deficits when compared to control mice.
Cheng W. et al. (2019) [49]	Wistar neonates male rats divided into four groups (*n* = 25 for each group): (a) control group, (b) neonatal isolation (NI) (c) single-prolonged stress (SPS) (d) NI+SPS group	-Plasma CORT levels; hippocampal and amygdala GR expression; Synapsin1 protein; PSD95; NLG proteins-1 and -2	NI between pnd2–pnd9 for 1 h per day	SPS on pnd56: 2 h of restraint, followed by 20 min of forced swim, followed by exposure to ether vapor leading to loss of consciousness	Plasma CORT response to SPS was significantly greater in the NI+SPS group compared with NI rats; GR immunoreactivity was higher in the dentate gyrus of the hippocampus and lower in basolateral amygdala (BLA) for the NI+SPS group compared to the SPS group; Synapsin1 reduced in the BLA and the hippocampal dentate gyrus of NI+SPS-group when compared with SPS-group rats; PSD-95 *hippocampal* expression and the ratio of NLG-1/-2 in rats of the NI+SPS group was *significantly higher* than that of rats in the SPS group; Finally, NI +SPS exacerbated the increased anxiety levels and impaired spatial memory than NI or SPS alone.

ACTH = pituitary adrenocorticotropic hormone; AEA = endocannabinoid anandamide; 2-AG = 2-arachidonoyglycerol; Allo = allopregnanolone; BDNF = Brain Derived Neurotrophic Factor; BLA = basolateral amygdala; CB = endocannabinoid system; CB1R = cannabinoid receptors type 1; CBG = corticosterone binding globulin; CORT: corticosterone; CRH = corticotrophine releasing hormone; dlBNST = dorsolateral bed nucleus of stria terminalis; e-CB = endocannabinoid system; e-FAAH = Fatty-acid amide hydrolase of anandamide; FKBP51 = FK506 binding protein 51; GABAergic = gamma-aminobutyric acidergic; HOME-HAN = repeated handling of brief maternal separation in home environment with al pups together; HOME-SEP: repeated maternal separation in home-environment, with the pups remaining together; IL-1β = proinflammatory interleukin 1β; IFNγ = proinflammatory interferon-γ; MS = maternal separation; mPFC = medial prefrontal cortex; mGR = membrane glucocorticoid receptors; mMR = membrane mineralcorticoid receptors; NI = neonatal isolation; NLG1,NLG-2 = neuroligin proteins 1–2; NOVEL-HAN = repeated handling of daily brief (15 min instead of 8 h) MS in the in a novel-environment individually; NOVEL-SEP = repeated MS, pups were individually housed in a novel-environment; PEA = N-palmitoylethanolamine; pnd = postnatal day; POW = Prisoner of War; 5aRI = 5aReductase Type I; PSD95 = postsynaptic density protein 95; P75NTR = signaling receptor of ProBDNF; RUS = repeated unpredictable stress; SBSS = selective brain steroidogenic stimulant; SSRI = Selective Serotonine Reuptake Inhibitor; SPS = single-prolonged-stress; TL = telomere length; TMT = Trimethylthiazoline, component of fox urine, URB597 = FAAH inhibitor.
